# Implementation of a novel primary care pathway for patients with severe and enduring mental illness

**DOI:** 10.1192/pb.bp.116.055830

**Published:** 2017-12

**Authors:** Frank Röhricht, Gopal Krishan Waddon, Paul Binfield, Rhiannon England, Richard Fradgley, Lise Hertel, Paul James, Judith Littlejohns, David Maher, Matthew Oppong

**Affiliations:** 1 East London NHS Foundation Trust; 2 University of Essex; 3 University of Bolton; 4 NHS City & Hackney Clinical Commissioning Group; 5 South East Mental Health Commissioning Network; 6 NHS Tower Hamlets Clinical Commissioning Group

## Abstract

**Aims and method** New collaborative care models with an emphasis on primary care are required for long-term management of patients with severe and enduring mental illness (SMI). We conducted a descriptive evaluation of clinical outcomes of the first 3 years of a novel enhanced primary care (EPC) service. Data from 2818 patients and staff survey results were analysed.

**Results** 2310 patients were discharged to EPC (508 not assessed as clinically suitable or patients/general practitioners declined the transfer); mean length of stay with secondary care service of the cohort was 9.8 years (range 0–24). 717 patients (31%) have been discharged to primary care only out of the EPC services and 233 patients (10%) have been transferred back to secondary care. Patient and staff satisfaction with the new EPC model was high. No severe untoward incidents were recorded.

**Clinical implications** The data suggest that EPC can be safely provided for a significant proportion of patients with SMI, who traditionally received long-term secondary care support. The novel EPC model can be utilised as a template for the provision of cost-effective, recovery-oriented and non-stigmatising care in the community.

Mental healthcare for the majority of patients with severe and enduring mental illness (SMI) was historically provided predominantly by specialist secondary care services in the UK. Hereby, most patients with long-term chronic conditions received open-ended health and social care support and relatively few were discharged back to primary care for ongoing case management. More recently, patients with SMI are increasingly considered for clinical management by less specialised, generic primary healthcare services and in collaborative care models.^1–3^ Most of these schemes have not been robustly evaluated through clinical and controlled trials. There is however good evidence to suggest that collaborative chronic care models foster patient self-management and can improve mental and physical outcomes for patients with mental health problems.^3^

In England, the introduction of clinical commissioning groups (CCGs) in 2013^4^ and the significant public spending savings plan with annual targets for all National Health Service (NHS) provider organisations^5^ resulted in a nationwide review of mental healthcare provisions and piloting of novel care pathways. Secondary mental healthcare providers have started to reduce the number of cases held by specialist community mental health teams, including assertive outreach and community rehabilitation teams. The NHS policy paper *No Health Without Mental Health*^6^ emphasised the need for integration of care for physical and mental health needs; provider and commissioner organisations therefore started to engage in various local service redesign projects. In East London, enhanced primary care (EPC) services for patients with SMI were developed and implemented in 2012, following a whole system review which attempted to address the issue of plateauing resources. The overall aim of the EPC service is to foster recovery of patients with SMI, to safely discharge them to primary care settings that attend to their mental healthcare needs. Three years after its launch, clinical outcomes of the novel care pathway for patients with SMI, patient/staff satisfaction were evaluated and are reported in this paper.

## Method

### Data collection and analysis

This evaluation is based on a retrospective data analysis of routinely collected data on all the patients referred to and/or fully discharged from secondary care to the EPC service since its inception (reporting period from 1 August 2012 to 31 July 2015). Electronic clinical data-sets included: total number of identified/discharged patients; number of patients who refused to be transferred to the new service and where either general practitioners (GPs) or consultant psychiatrists assessed patients as not suitable; diagnoses and ICD-10^7^ codes, gender, ethnicity, duration of service provided by secondary care, number of admissions to hospital; number of patients re-referred to secondary care or readmitted/relapsed analysis of main characteristics of this subgroup of patients.

In addition we conducted an analysis of available information collected regarding staff satisfaction (semi-structured interviews and questionnaires) and summarised data from a patient satisfaction survey (Client's Assessment of Treatment Scale^8^) for the new service from one of the three localities (Newham).

### Description of the new EPC service

The model was developed in partnership between East London NHS Foundation Trust and the three East London CCGs (Newham, City & Hackney, Tower Hamlets) across primary/secondary care teams by interdisciplinary working groups; those involved were people participation leads, GPs, consultant psychiatrists, senior community psychiatry nurses, psychologists and social work leads. The initial target was defined as 300 patients per year per locality. The EPC care pathway allows providing mental healthcare flexibly according to patients' needs and to step up (secondary mental health services) or step down (generic primary care) in a seamless way without administrative hurdles.

### EPC care pathway elements

Elements of the EPC care pathway were as follows:

- Regular GP reviews (in addition to quality and outcome requirements), the development of a recovery care plan, practice nurse administration of depot medication, and specific assessment of risk factors for physical illness, signposting into healthy lifestyle services.
- Enhanced support to primary care from consultant psychiatrists with regular practice-based mental health multidisciplinary review meetings.
- Training and education to GPs on managing SMI in primary care, and for practice nurses on psychopharmacology and therapeutic depot administration.
- EPC mental health teams consisting of registered mental nurses, employed by East London NHS Foundation Trust but working within general practice, to support discharge into the EPC and provide recovery-oriented support to patients on an ongoing basis.

The philosophy of the service operationally distinguishes clearly between:

- specialist acute and rehabilitation treatment for patients with severe or complex needs and/or those identified as presenting with significant risks to self or others associated with their illness (continued to be provided by secondary care services); and
- long-term condition management aiming to provide monitoring and maintenance support for patients with stable chronic SMI (provided by the new EPC teams).

Inclusion criteria for the EPC transfer were defined as follows:

- Aged 18 years or older, resident in one of the three East London boroughs and currently under the care of secondary care services.
- Established diagnosis of an SMI that would warrant their inclusion on the practice severe and enduring mental illness (SEMI) register.
- Identified care needs above those that would ordinarily be provided for under normal primary care, either medical or social needs, that require additional support.
- Patient agrees to the support offered via the EPC clinic.
- Sustained clinical progress with less frequent support from secondary care and no identified need for specialist interventions and treatments.
- Last acute psychiatric hospital admission more than 12 months ago, no current risks to self or others identified; patient is well-established on a medication regime and requires minimal assistance with concordance, but does require regular monitoring and review.
- Patient has settled accommodation, is able to meet their own basic living needs.
- Patients requiring lithium prescribing or depot injections in primary care are included.

### Teaching, training and supervision

Primary care liaison nurses (PCLNs) of the EPC teams were recruited from secondary care services and all had experience in providing mental healthcare to patients with SMI. A list of essential competencies, skills and experiences were identified as significant requirements for the recruitment of the EPC workforce and included the following areas: history taking/mental state examination, engagement skills and basic knowledge of principles pertaining to a positive therapeutic relationship, risk assessment and management, recovery care and social psychiatry, and psychiatric emergencies. Additional training was offered to PCLNs.

The provision of the EPC service is supported through weekly team meetings, monthly supervision sessions, and six monthly appraisal/reviews, aiming to maintain and update knowledge and skills.

The three localities agreed on mandatory training requirements for primary care staff as part of the EPC contract with their primary care practices and the EPC team provided training for staff in GP practices including mental health awareness training for practice reception staff and depot training for nurses.

Subsequently, in 2015 a website with a wide range of teaching and self-learning materials for all primary care staff was developed with funding from Health Education England North Central and East London, launched into public domain in 2016 (http://primary-mentalhealth-care.elft.nhs.uk).

### Service aims and expected outcomes

- To support patients to achieve their recovery goals through a process of joint planning that places patients at the centre.
- To empower people to self-manage their own recovery journey and reach a position where they can reduce their contact with mental health services.
- To mark the recovery journey by recognising achievements while being transferred to receive care in EPC and at the point of discharge from EPC.
- To improve the patient experience and outcomes through enhanced multidisciplinary team working that addresses mental health, physical health and social need as part of an integrated approach.
- To improve patient experience and outcomes through the provision of care in a normalised setting, close to home and to assist the navigation of patients towards resources that supports their recovery.
- To enable the development of capacity, confidence and competence in relation to mental health treatment and care in the primary care workforce.

The service elements were developed accordingly, centred on a ‘My Recovery Plan’ and associated ‘My Safety Plan’ documents for time-limited EPC interventions according to recovery goals set by the patient (with a recommended duration of up to a year after which the patient is supposed to be discharged into generic primary care services). The time-limited nature of the service was to be made clear to the patients at the outset and the service provides a fast-track option back to secondary care services if needs change.

### Team composition

The service is predominantly delivered by psychiatric PCLNs. The three localities agreed on different staffing compositions according to local variations of service needs and/or perceived requirements in respect of multidisciplinary inputs from health professionals.

In Newham, psychiatrist input is provided by one consultant with protected time in the job plan and by catchment area consultants as required based on a sectorised/practice-aligned service model; in Tower Hamlets consultants with a primary base in general practice deliver psychiatric expertise; and in City & Hackney a model with a dedicated EPC service consultant was established.

Following a 12-month review the team of healthcare professionals was complemented by a group of peer support workers, who provide patients with emotional and practical support as they approach discharge from secondary care services. All the peer supporters have personal experience of in-patient and community mental healthcare and support patients flexibly based on their own experiences of recovery. A summary of the current team structures and allocated posts is provided in Table 1.

**Table 1:** Staffing composition in enhanced primary care (EPC) teams in each locality^a^

Directorate	Total WTE per team	Admin Band 4	Occupational therapist	Clinical psychologist	Nurse Band 6	Nurse Band 7	Consultant	Peer/ support worker Band 3/4	Service manager Band 8a	Team case-load capacity
CH original	10.6					8.0	1.6		1.0	300

CH current	16.3	1.0	0.2	0.2		6.0	1.0^b^	7.0	1.0	720

NH original	9.1				4.0		0.1	4.0	1.0	300

NH current	14.0				4.0		0.5^c^	8.0	1.0	600

TH original	6.4				6.0		0.4			250

TH current	18.0	1.0			6.0	4.0	1.5^d^	4.0	1.0	700

Trust total current	48.3	2.0	0.2	0.2	10	10	3.0	19.0	3.0	2020

CH, City & Hackney; NH, Newham; TH, Tower Hamlets; WTE, whole time equivalent.

a. Original and current from 2016. Phased increase in size of service with new investment following a service review in 2014–2015.

b. Consisting of each of the four current primary care consultants devoting 2.5 sessions a week to the EPC service and primary care liaison.

c. Consisting of time dedicated to EPC and primary care liaison by the four assessment and brief treatment consultants providing support to practices and one consultant with 0.1 WTE leading on EPC.

d. Consisting of a dedicated consultant providing two sessions a week to provide clinical support to the EPC team and the Compass Primary Care Psychology service, and each of the community mental health team consultants providing one session a fortnight to supporting the primary care practices to which they are aligned.

EPC provides an open general advice service to GPs to assist in the treatment of patients that have been discharged from EPC and secondary care. The arrangements vary between the three East London boroughs but all include case-based discussions between GPs and consultant psychiatrists during regular multidisciplinary clinical meetings at primary care level.

## Results

### Service activity summary

As per 31 July 2015, the three East London EPC teams provided care for 1370 patients. Since August 2012 the services considered in total 2810 patients, of which 480 were not proceeded with because the patients declined transfer (*n* = 149), the GP declined the transfer (*n* = 90) or the secondary care eventually decided the transfer was not clinically appropriate (*n* = 241). Therefore, 2330 patients have received an active service from EPC services since their inception. In total, 717 patients were transferred to primary care only from EPC with variations across teams. The total number of patients discharged from EPC to primary care alone has significantly increased beyond the reporting period of this service evaluation due to changes in operational policy and as a result of the teaching and training efforts to upskill GPs, resulting in increased throughput.

All referral and case-load data with developments to 30 October 2016 are summarised in Table 2.

**Table 2:** Total referral and case-load activity for enhanced primary care (EPC)

	At 31 July 2015	At 29 February 2016	At 30 October 2016
Active case-load, *n*			
City & Hackney	510	547	633
Newham	485	557	610
Tower Hamlets	375	473	618
Total *n*	1370	1577	1861

Referrals considered, total *n*			
EPC – Trust wide	2810	4082	5286

Refused/not suitable, *n*			
EPC – Trust wide	480	576	633

Received EPC service, *n*			
City & Hackney	955	1317	1635
Newham	787	1186	1448
Tower Hamlets	588	1003	1570
Total *n*	2330	3506	4653

EPC transfer to primary care, *n*			
City & Hackney	384	675	939
Newham	184	465	705
Tower Hamlets	149	385	594
Total *n*	717	1525	2238

Transfer back to secondary care, *n*			
City & Hackney	65	95	177
Newham	124	164	227
Tower Hamlets	48	94	160
Total *n*	233	353	564

### Patient characteristics

The majority of patients referred to EPC had a significant history of SMI with an average (mean) duration of care provided under care programme approach (CPA) standards by secondary care services (community mental health teams) of 9.7 years (range 0–24). The mean age of patients was 45.7 years (range 18–65; 12.1% 18–30 years and 77.3% 30–60 years); 54% of patients were female and 46% were male. Overall, 47.4% were single/living alone, 26.3% were married/civil partner and 11.6% were separated/divorced/widowed/surviving civil partner. Given the high percentage of Black and ethnic minorities living in East London, the distribution of ethnicity across the sample reflects the diversity: Asian or Asian British 24%, Black or Black British/African–Caribbean 19.8%, White British or other White 38.5%, other ethnic groups 17.8%.

The main diagnoses of patients were: schizophreniform or other psychotic disorders (ICD-10 codes F20–29) 37.2%, mood (affective) disorders (F30–39) 32.1%, anxiety/stress-related/somatoform and other non-psychotic mental disorders (F40–48) 11% and disorders of adult personality and behaviour (F60–69) 4.1%.

According to Department of Health guidance^9^ the main cluster codes on transfer to EPC were: cluster 10–13: 48.9% (11: 19.4%; 12: 21.5%; 13: 7.1%); cluster 4–7: 26.1%. The number of patients referred back to secondary care due to clinical issues (relapse concerns) was 237 (City & Hackney *n* = 65, Newham *n* = 124, Tower Hamlets *n* = 48).

### Feedback from patients

Both EPC staff reports and results from questionnaire surveys suggest that the vast majority of patients regarded the new service arrangements as both helpful and adequate according to their needs. We conducted a more detailed survey in one of the three localities (Newham), using the structured Client's Assessment of Treatment Scale. Results from 126 patients who completed the survey (mean age 49.2 years, range 26–71; 66 female, 60 male) are indicative of comparatively high levels of patient satisfaction (most scores across the group rated with a mean of 8–9 out of 10) (Table 3).

**Table 3:** Results from 126 patients who completed the Client's Assessment of Treatment Scale

	Mean	Range	s.d.
Do you believe you are receiving the right treatment/care for you here?	8.8	4–10	1.6

Does your general practitioner understand you and is she/he engaged in your treatment/care?	8.4	1–10	1.9

Does your named nurse understand you and is she/he engaged in your treatment/care?	9.0	2–10	1.5

Are relations with other staff members here pleasant or unpleasant for you?	8.5	0–10	2.0

Do you believe you are receiving the right medication for you?	8.9	0–10	1.7

Do you believe the other elements of treatment/care here are right for you?	9.4	4–10	1.3

Do you feel respected and regarded well here?	9.0	4–10	1.5

Has treatment/care here been helpful for you?	9.0	4–10	1.4

### Feedback from GPs

GPs across all three localities engaged very well with the three EPC teams and expressed high levels of satisfaction; they acknowledged that the EPC service improved care for their patients. A brief survey questionnaire distributed to 61 GP surgeries in Newham was returned by 52 GPs. All but two GPs stated that the EPC helped to change their perception of/and relationship with mental health services.

Another GP survey was conducted in Tower Hamlets and revealed the following feedback (first figure 6 months after service implementation based on 61 responses (from 36 surgeries), second figure 1 year later based on 23 responses); this survey indicates that the EPC model contributed to developing GP's skills and knowledge of psychotropic prescribing (Very confident 3.3/13%, Confident 44.3/47.8%, Neutral 33.4/34.8%, Not confident 18.0/4.4%). In addition, satisfaction rates with practice-based multi-disciplinary meetings as well as the network-linked PCLNs was largely positive and increased over time.

We conducted a subgroup analysis of patients from Newham EPC who were re-referred to secondary care from EPC due to a relapse (significant increase in symptoms) of their mental disorder or other reasons; *n* = 124 out of 787, 15.8%.

Relapse due to a range of stressors (iatrogenic, non-adherence, etc.) was *n* = 69; relapse with acute admission to hospital, *n* = 8; and non-engagement and requests to be discharged from GP, *n* = 9. Requests for medication review by secondary services/GP referred back: *n* = 26; social circumstances: *n* = 4; and patient demanding to be referred back to consultant: *n* = 3.

Only for 3 out of 124 re-referred patients with a change in prescribed dose of medication were identified, all others had been on stable medication as per discharge plan from secondary care. The number of EPC face-to-face contacts for this group varied from 0 to 8, most patients had been seen on 1–3 occasions by their PCLN. The diagnostic codes, PCLN clinics and GP surgeries were equally distributed across this group.

## Discussion

The data-set considered for this service evaluation comprised of a large sample of over 2000 patients with predominantly chronic severe mental illness (schizophreniform, psychotic or severe affective disorder, care clusters 10–13 and 4–7) and the observation period of 3 years seems adequate to allow for a critical appraisal of performance data. The overall results from this service evaluation suggest that a significant proportion of patients with SMI, who were traditionally seen long term with open-ended care plans in secondary care, can be successfully discharged to enhanced primary mental healthcare services. This is even more so significant given the fact that prior to transfer of care, patients had been receiving specialist mental health services for on average of nearly 10 years. The number of relapses and re-referrals to secondary care services has been low, even though the overall referral rate to EPC has risen. A significant number of patients who received EPC services are now supported by primary care alone.

The success of this novel care pathway is based on very close collaboration between primary and secondary care health professionals and service characteristics that provide seamless care across boundaries: all PCLNs were employed through secondary mental health services and mostly recruited from existing mental health teams, which enabled them to provide clinical expertise into the new service – quick access to secondary care for crisis management was built into the service structure.

Only about 14% of patients were not taken into EPC clinic care following the initial referral and this is indicative of a carefully conducted and initially conservative selection process, also taking patient preferences into account. Patient feedback was very positive and no severe untoward incidents occurred during the observational period.

Although mental healthcare services for patients with SMI have traditionally been regarded as too specialised for primary care, most patients regard primary care provisions as a significant milestone in their recovery journey.^10^ The care pathway development was conducted based on a much clearer distinction between elements focusing on supporting people to maintain stability and monitor symptoms versus elements providing active recovery-oriented treatment. This allowed refocusing of specialist services and deconstructing the ‘shifted out-patient clinic’ model,^11^ essentially a replacement model, which does not provide opportunities for enhanced linkage and face-to-face consultations between the primary care physician and the psychiatrist.^12^ By contrast, the consultation-liaison collaboration model provides regular face-to-face contact between the psychiatrist or mental health worker and the GP.^13^

The survey results suggest that the support primary care doctors receive from consultant psychiatrists is a vital part of the scheme. The precise arrangements vary across the three boroughs but each primary care practice has an aligned consultant who visits the surgery regularly, is available for advice especially on potential referrals to secondary care and who assists the surgeries to become more mental health sensitive and informed.

The main difficulties with the new care pathway identified in the context of this service evaluation are related to the wider context of recovery-focused care with an emphasis on integration with mainstream community services, such as employment, training and leisure activities. Depending on pre-existing skill and knowledge base within each of the participating GP surgeries, the quality of mental state monitoring and therapeutic engagement is likely to vary significantly. More emphasis must therefore be given towards developing robust and ongoing teaching and training curricula for primary care practitioners. Another significant challenge is the variation in access to psychological therapy services and social care from locality to locality depending on the level and specification of integrated care pathways. This is a crucially important issue for the success of EPC services, safeguarding against compromising the quality of care.

A further significant increase of the number of people who experience a mental health problem in England has been predicted (i.e. 14.2%, from 8.65 million in 2007 to 9.88 million in 2026) as a result of population growth.^14^ More research of innovative and collaborative schemes for high-quality cost-effective mental healthcare is required, assessing the impact of working across primary and secondary care.^15^

The promotion of psychological resources and capabilities at a family and community level to support people experiencing mental ill health appears to be a promising complementary strategy for both primary and secondary prevention. Last but not least there seems to be a real case to extend the role of district nurses, to strengthen the role of GP champions in mental healthcare^16^ and to involve patients as teachers in interprofessional learning as already pointed out by Lester *et al*^17^ in their discussion paper on integrated primary mental healthcare more than 10 years ago.

### Limitations and outlook

This is a retrospective analysis of routinely collected data for service evaluation, not a formal research study. Patients were identified by their secondary care clinicians as potentially suitable for transfer to the EPC clinic and there was no control condition. The Client's Assessment of Treatment Scale satisfaction scores and relapse indicator analysis was only available for one of the three localities and only a subgroup of about 25% of patients open to the EPC service completed the survey.

Empirical research is needed to establish detailed patient characteristics as predictors for successful transfer of care. Longer-term and controlled follow-up studies are required to establish care quality and effectiveness issues across various components of the health and social care pathway (e.g. social inclusion, subjective quality of life, psychopathological symptom levels) following discharge from secondary care services, compared with continuing specialist treatment. It will be important to assess differences between inner-city and more rural areas to establish as to whether the claim, that the delivery of mental healthcare in primary settings is ‘more accessible, affordable and acceptable for the population’^18^ can be substantiated.
